# Clinicopathologic Comparison of High-Dose-Rate Endorectal Brachytherapy versus Conventional Chemoradiotherapy in the Neoadjuvant Setting for Resectable Stages II and III Low Rectal Cancer

**DOI:** 10.1155/2012/406568

**Published:** 2012-07-08

**Authors:** Jessica A. Smith, Aaron T. Wild, Aatur Singhi, Siva P. Raman, Haoming Qiu, Rachit Kumar, Amy Hacker-Prietz, Ralph H. Hruban, Ihab R. Kamel, Jonathan Efron, Elizabeth C. Wick, Nilofer S. Azad, Luis A. Diaz, Yi Le, Elwood P. Armour, Susan L. Gearhart, Joseph M. Herman

**Affiliations:** ^1^Department of Radiation Oncology & Molecular Radiation Sciences, Sidney Kimmel Comprehensive Cancer Center, Johns Hopkins University School of Medicine, Baltimore, MD 21205-2196, USA; ^2^Department of Pathology, Sidney Kimmel Comprehensive Cancer Center, Johns Hopkins University School of Medicine, Baltimore, MD 21205-2196, USA; ^3^Department of Radiology, Johns Hopkins University School of Medicine, Baltimore, MD 21205-2196, USA; ^4^Department of Surgery, Sidney Kimmel Comprehensive Cancer Center, Johns Hopkins University School of Medicine, Baltimore, MD 21205-2196, USA; ^5^Department of Oncology, Sidney Kimmel Comprehensive Cancer Center, Johns Hopkins University School of Medicine, Baltimore, MD 21205-2196, USA

## Abstract

*Purpose*. To assess for differences in clinical, radiologic, and pathologic outcomes between patients with stage II-III rectal adenocarcinoma treated neoadjuvantly with conventional external beam radiotherapy (3D conformal radiotherapy (3DRT) or intensity-modulated radiotherapy (IMRT)) versus high-dose-rate endorectal brachytherapy (EBT). *Methods*. Patients undergoing neoadjuvant EBT received 4 consecutive daily 6.5 Gy fractions without chemotherapy, while those undergoing 3DRT or IMRT received 28 daily 1.8 Gy fractions with concurrent 5-fluorouracil. Data was collected prospectively for 7 EBT patients and retrospectively for 25 historical 3DRT/IMRT controls. *Results*. Time to surgery was less for EBT compared to 3DRT and IMRT (*P* < 0.001). There was a trend towards higher rate of pathologic CR for EBT (*P* = 0.06). Rates of margin and lymph node positivity at resection were similar for all groups. Acute toxicity was less for EBT compared to 3DRT and IMRT (*P* = 0.025). Overall and progression-free survival were noninferior for EBT. On MRI, EBT achieved similar complete response rate and reduction in tumor volume as 3DRT and IMRT. Histopathologic comparison showed that EBT resulted in more localized treatment effects and fewer serosal adhesions. *Conclusions*. EBT offers several practical benefits over conventional radiotherapy techniques and appears to be at least as effective against low rectal cancer as measured by short-term outcomes.

## 1. Introduction

Colorectal cancer is the 3rd most common malignancy among both men and women in the United States [1]. Approximately thirty percent of colorectal adenocarcinomas occur in the rectum, totaling approximately 40,290 newly diagnosed cases per year [2]. There are two main goals of treatment for rectal adenocarcinoma, with the first being complete resection to minimize the risk of recurrence and the second being sphincter preservation in order to maintain normal evacuative function. The current standard of care for patients with stage II-III resectable adenocarcinoma of the rectum is neoadjuvant chemoradiation consisting of 5-fluorouracil- (5-FU-) based chemotherapy and external beam radiation using intensity modulated (IMRT) or 3D conformal (3DRT) radiotherapy techniques. Chemoradiation is followed by total mesorectal excision (TME) with either a lower anterior resection (LAR; sphincter preserving) or an abdominoperineal resection (APR; nonsphincter preserving) and adjuvant FOLFOX chemotherapy [3]. The time frame of conventional neoadjuvant therapy is 5-6 weeks of concurrent chemoradiation followed by a 6–8 week recovery window, then surgical resection. 3DRT or IMRT techniques are considered the standard of care, with a total dose of 50.4 Gy given over 28 fractions.

**Figure 1:**
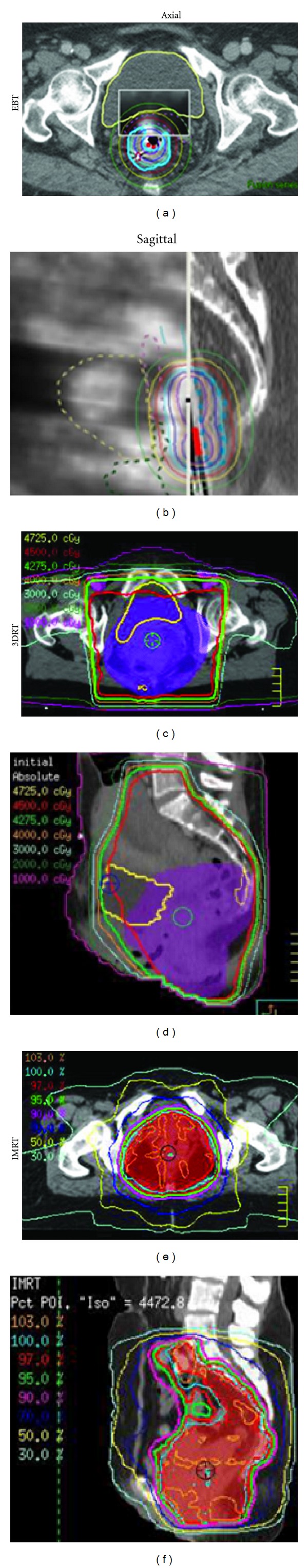
Representative slices from each of the three radiation plan types taken from a similar level in the pelvis. EBT can be seen to achieve a high dose to the tumor while exposing markedly less normal tissue volume to ionizing radiation as a result of rapid dose fall off from the point source. Top row: in axial (a) and sagittal (b), slices from an EBT plan, the 100% (light blue), 95% (red), 50% (yellow), and 30% (green) isodose lines are shown and the tumor perimeter is contoured (thick light blue line) as well as the bladder perimeter (thick yellow line in axial image, dotted yellow line in sagittal image). Middle row: in axial (c) and sagittal (d) slices from a 3DRT plan, the 100% (red), 95% (bright green), 89% (orange), 67% (gray), 44% (dark green), and 22% (fuchsia) isodose lines are shown, and the planning target volume receiving the full radiation dose around the tumor is indicated (purple shading) as well as the bladder perimeter (yellow). Bottom row: in axial (e) and sagittal (f) slices from an IMRT plan, the 100% (light blue), 97% (red), 95% (green), 90% (fuchsia), 70% (royal blue), 50% (yellow), and 30% (gray) isodose lines are shown, and the planning target volume receiving the full radiation dose around the tumor is indicated (red shading).

Preoperative external beam radiation has been shown to increase pathological response rates and reduce the risk of local recurrence [3], but it is also associated with an increased risk of therapy-induced side effects and increased morbidity [4]. These acute toxicity events may lead to treatment breaks, compromising the efficacy of treatment and delaying surgery [3]. In an attempt to reduce treatment-related toxicity, high-dose-rate endorectal brachytherapy (EBT) was developed as an alternative neoadjuvant option for locally advanced low rectal cancer. This technique has been previously described as monotherapy for the treatment of prostate, cervical, esophageal, and buccal mucosal tumors [5–7]. EBT is a radiotherapy technique that allows for delivery of a focused high dose of ionizing radiation at the mucosal surface directly overlying the tumor while avoiding injury to surrounding normal tissues. Rapid dose fall off from the iridium-192 point source and the lack of external radiation beams that must pass through the normal pelvic tissues to reach the tumor combine to minimize dose to normal surrounding structures such as the femoral heads, bowel, bladder, and reproductive organs compared to conventional radiotherapy techniques (Figure 1). In high-dose-rate brachytherapy, the radioisotope is inserted for a brief period of time (approximately 15 minutes for EBT) to deliver the required dose and then withdrawn from the body, as opposed to low-dose-rate brachytherapy where a radioactive source is left implanted in the patient. EBT is given in 4 fractions of 6.5 Gy, for a total dose of 26 Gy. The treatment consists of 4 days of radiation treatment alone followed by a 6–8 week recovery window, then surgical resection and adjuvant chemotherapy. Investigators at McGill University have achieved excellent tumor regression with over 29% of patients having a complete pathologic response at surgery [4, 6, 8]. The response rates of EBT are similar if not superior to those achieved with conventional neoadjuvant chemoradiation, for which the associated pathologic CR rate ranges from 8% to 19% [9–14]. The potential benefits of EBT for patients include the short duration of therapy, the seemingly high response rate reported thus far, and the avoidance of concurrent neoadjuvant systemic chemotherapy and its associated toxicity.

At this point, only one group has published data on patients with resectable rectal adenocarcinoma who were treated with EBT. Herein, we analyze the preliminary outcomes obtained with EBT and compare them to patients receiving conventional neoadjuvant chemoradiation (3DRT/IMRT) at our institution. The primary goal of this study, therefore, is to compare radiologic, pathologic, and short-term clinical outcomes between EBT and conventional radiation techniques in the neoadjuvant setting.

## 2. Materials and Methods

### 2.1. Patient Selection

From 2010–2012, 7 patients with locally advanced low rectal adenocarcinoma (within 12 cm of the anal verge) were enrolled in a prospective study of neoadjuvant EBT (NCT01226979) at Johns Hopkins Hospital. Patients were required to meet the following inclusion criteria: greater than 18 years of age, histologically confirmed adenocarcinoma of the rectum, able to undergo local staging by MRI and/or EUS demonstrating a T2N1 or T3N0-1 tumor, and ECOG performance status of 0 or 1. Patients were excluded if they had tumors greater than 12 cm from the anal verge, metastatic disease at time of enrollment, positive inguinal or iliac lymph nodes on MRI, PET, or EUS, concurrent malignancy, bulky tumors that would not allow application of the endorectal probe, or previous pelvic irradiation. For comparison, historical controls were obtained by identifying all patients with stage II-III rectal adenocarcinoma who received conventional neoadjuvant chemoradiation with IMRT or 3DRT at our institution from 2008–2012 and went on to surgical resection.

### 2.2. Clinical Outcomes

 Clinical data for patients treated with EBT was gathered prospectively as part of the trial protocol. To gather data for historical controls, retrospective chart review was performed using the electronic patient record (EPR) system after approval by the Institutional Review Board. For all patients, toxicity was evaluated using the National Cancer Institute (NCI) Common Terminology Criteria for Adverse Events (CTCAE) version 4.0.

### 2.3. Neoadjuvant Therapy

Patients in the EBT group were treated with 4 consecutive daily fractions of 6.5 Gy targeted to the tumor and mesorectal lymph nodes without concurrent chemotherapy. Each fraction was delivered over approximately 15 minutes using a flexible silicone intracavitary applicator (OncoSmart, Nucletron, Veenendaal, The Netherlands) positioned in the rectum using fluoroscopic guidance and a microSelectron high-dose-rate iridium-192 remote afterloading system (Nucletron) as described by Vuong et al. [4]. Treatment planning was performed using the Oncentra brachytherapy planning system (Nucletron). Patients in the 3DRT and IMRT groups received 28 daily (Monday through Friday) fractions of 1.8 Gy over a period of 5 to 6 weeks (total dose of 50.4 Gy) with concurrent oral 5-FU.

### 2.4. Surgical Resection

At the initial assessment for all patients, surgery was preplanned according to the standard of care to take place from 6 to 8 weeks following completion of neoadjuvant therapy. All patients were able to undergo surgical resection after neoadjuvant therapy consisting of total mesorectal excision (TME) accomplished as part of a lower anterior resection (LAR) or abdominoperineal resection (APR) procedure. When possible, LAR was performed in preference to APR so that the anal sphincter and normal evacuative function could be preserved.

### 2.5. Radiologic Assessment

MRI images of the pelvis, including high resolution T2 weighted images of the rectum, were acquired in 3 planes both prior to and following neoadjuvant therapy (see Tables 1 and 3 for specific timing of MRI imaging in relation to radiotherapy). Each study was evaluated by a blinded gastrointestinal radiologist. At each time point, the tumor was measured in 3 dimensions (maximum length and width on axial cross-section as well as maximum craniocaudal extent in the coronal or sagittal plane). These measurements were used to generate volume estimates for each tumor using the formula for volume of an ellipsoid (*V* = *π*/6 × *A* × *B* × *C*, where  *A*,  *B*, and  *C*  are the maximum tumor diameters along the *x*-,  *y*-, and  *z*-axes). Changes in tumor volume after neoadjuvant therapy were calculated and tumor response rates were assessed using the sum of the maximum tumor diameters according to RECIST. Contrast enhanced T1 weighted MRI images obtained pre- and postneoadjuvant therapy were used to delineate and measure any abnormal mesorectal lymph nodes as well as any suspicious appearing lymph nodes in the inguinal or iliac chains measuring greater than 1 cm in diameter.

### 2.6. Pathologic Assessment

Pathologic tumor response was assessed by postoperative evaluation of TME specimens. After macroscopic examination of the surgical specimens, the entire tumor was submitted along with representative sections of the surgical margins, surrounding bowel, and dissected lymph nodes for formalin fixation. After fixation, the tissue was paraffin embedded and serially cut into 5-micrometer sections. Hematoxylin and eosin (H&E) stained sections were examined microscopically. Final pathologic stage, tumor size, nodal status, metastatic disease, and documentation of treatment effect were recorded. If present, lymphovascular invasion and positive surgical margins were also noted. Tumors considered to be completely responsive to preoperative therapy had no histologic evidence of residual carcinoma. Tumors with microscopic disease or large areas of residual carcinoma were considered partially responsive or nonresponsive to treatment, respectively. Slides from 5 randomly selected patients from each treatment group were rereviewed by a blinded pathologist to evaluate for any differences in radiation-induced treatment effects between the EBT, 3DRT, and IMRT groups.

### 2.7. Statistical Analysis

Statistical analyses were performed with IBM SPSS Statistics software, version 19 (International Business Machines Corporation, Armonk, NY). Patient characteristics consisting of continuous and dichotomous variables were summarized using descriptive statistics. Comparison of proportions between two or more groups was performed using the Pearson chi-squared test. Comparison of means between two groups (usually the EBT group versus the 3DRT and IMRT patients combined) was performed using the nonparametric Mann-Whitney  *U*  test. Comparison of means among three or more groups (usually EBT versus 3DRT versus IMRT) was performed using a one-way analysis of variance (ANOVA). An alpha level of less than or equal to 0.05 was considered significant in all cases.

## 3. Results

### 3.1. Patient Characteristics

All patient characteristics are summarized in Table 1. The EBT, 3DRT, and IMRT groups consisted of 7, 14, and 11 patients, respectively. Median lengths of followup were 7 months for EBT, 15 months for 3DRT, and 12 months for IMRT. Demographic and baseline disease characteristics, including age, race, ECOG performance status, pre-RT carcinoembryonic antigen (CEA) level, pre-RT tumor volume, T stage, N stage, and tumor distance from the anal verge, were similar among the 3 groups (all *P* > 0.05; Table 1). There was, however, a difference in gender distribution between the 3 groups, with 100% of EBT patients being female compared to only 29% and 64% of the 3DRT and IMRT groups, respectively (*P* = 0.007).

### 3.2. Clinical Outcomes

Clinical outcomes of interest included time to surgical resection, change in CEA level after neoadjuvant therapy, acute toxicity, sphincter preservation, and postoperative complications; these outcomes are summarized in Table 2. Time elapsed from the start of neoadjuvant therapy to surgical resection was reduced by nearly half in patients who underwent EBT as opposed to 3DRT or IMRT (*P* < 0.001). This reduction is not unexpected given the shorter time course of EBT (4 days) compared to 3DRT or IMRT (5-6 weeks). More interestingly, however, the time elapsed from conclusion of neoadjuvant therapy to surgical resection was also reduced for patients who underwent EBT (*P* = 0.038), despite the fact that all surgeries were similarly planned to take place 6–8 weeks following completion of neoadjuvant therapy. All 3 groups demonstrated similar median reductions in CEA levels after neoadjuvant therapy (*P* = 0.36). Fewer patients experienced grade 1 or 2 acute toxicity in the EBT group than in the external beam group (*P* = 0.025). Grade 3 toxicity was rare, occurring in one patient from each of the 3 groups with all 3 incidents taking the form of proctitis. No grade 4 toxicity was reported. Rates of sphincter preservation and postoperative complications were similar among the 3 groups. Given the natural history of rectal adenocarcinoma, length of followup was not sufficient to perform informative analyses of survival and disease progression; however, preliminary results are given here to allow for comparison of EBT to conventional neoadjuvant therapy at the current length of followup. The rates of overall survival and local recurrence free survival at 6 months were 100% in all 3 groups. The rate of distant metastasis at 6 months was 0% for EBT, 7% for 3DRT, and 9% for IMRT. Thus, at a 6-month time point, EBT appears noninferior to conventional neoadjuvant chemoradiation using 3DRT or IMRT.

### 3.3. Radiologic Outcomes

**Figure 2:**
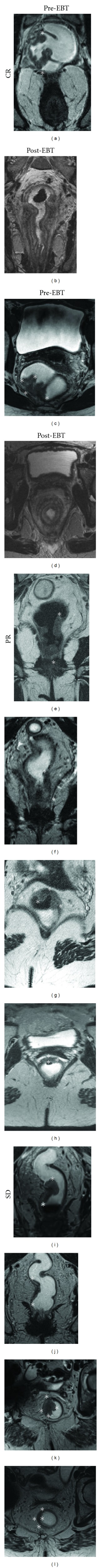
Representative pre- and posttreatment MRI slices from patients who underwent EBT and were found to have a complete pathologic response (pCR: defined as no residual tumor on histopathologic examination) at surgery. Although all 3 patients achieved a complete pathologic response, they demonstrated differing degrees of radiologic response on MRI according to RECIST, suggesting that degree of radiologic response does not necessarily predict for degree of pathologic tumor response. The longest tumor dimensions in 3 planes used for RECIST assessment are indicated by white asterisks. Scans represented in the top, middle, and bottom rows were obtained 39, 34, and 32 days following the completion of radiotherapy, respectively. Top row: coronal (a), (b) and axial (c), (d) MRI slices from a patient with a pCR who also demonstrated a radiologic complete response (CR); no residual tumor is visualized on post-EBT MRI (b), (d). Middle row: coronal (e), (f) and axial (g), (h) MRI slices from a patient with a pCR who demonstrated a radiologic partial response (PR) on post-EBT MRI (f), (h). Bottom row: coronal (i), (j) and axial (k), (l) MRI slices from a patient with pCR who demonstrated stable disease (SD) on post-EBT MRI (j), (l).

Radiologic outcomes of interest included change in tumor volume, tumor response rates analyzed according to RECIST, and change in mesorectal and pelvic nodal disease status over the course of neoadjuvant therapy. These outcomes are summarized in Table 3 for all patients in each group who had pre- and posttreatment MRI studies available (*n* = 7 for EBT, *n* = 10 for 3DRT, *n* = 7 for IMRT). All 3 groups showed a striking response to neoadjuvant therapy, with similarly marked reductions in tumor volume. Thus, EBT achieved a comparable degree of reduction in tumor volume as measured on MRI after only 4 days of treatment without chemotherapy as 3DRT and IMRT achieved over 5 to 6 weeks with concurrent 5-FU. Tumor response rates according to RECIST were also similar to the 3 treatment techniques (*P* > 0.05 for rates of CR, PR, SD, and PD, as summarized in Table 3). Identification of clinically significant (≥1.0 cm in longest dimension) lymph nodes on pre- and posttreatment MRI showed that the proportion of patients with radiologic mesorectal nodal involvement decreased over the course of neoadjuvant therapy in the EBT and 3DRT groups, but remained stable in the IMRT group. Clinically significant pelvic lymph nodes were identified on pretreatment MRI in 4 patients in the 3DRT group; in all 4 patients, these nodes remained clinically significant following treatment. One patient in the IMRT group was observed to have a pelvic lymph node prior to treatment, which subsequently resolved after chemoradiation. No patient in any group developed new pelvic nodal involvement over the course of neoadjuvant therapy. The degree of radiologic tumor response, however, did not appear to correlate with pathologic complete response (pCR), with more patients manifesting a pCR at surgery than were observed to have a complete radiologic response on post-EBT MRI (Figure 2).

### 3.4. Pathologic Outcomes

Pathologic outcomes of interest included tumor complete response rate, surgical margin status, lymph node involvement, and lymphovascular invasion; these outcomes are summarized in Table 4. There was a trend towards higher rate of complete pathologic response in patients who underwent EBT (43%) compared to the external beam group consisting of 3DRT and IMRT patients combined (12%) (*P* = 0.06). Rates of margin positivity, lymph node involvement, and lymphovascular invasion were similar among the three treatment groups.

**Figure 3:**
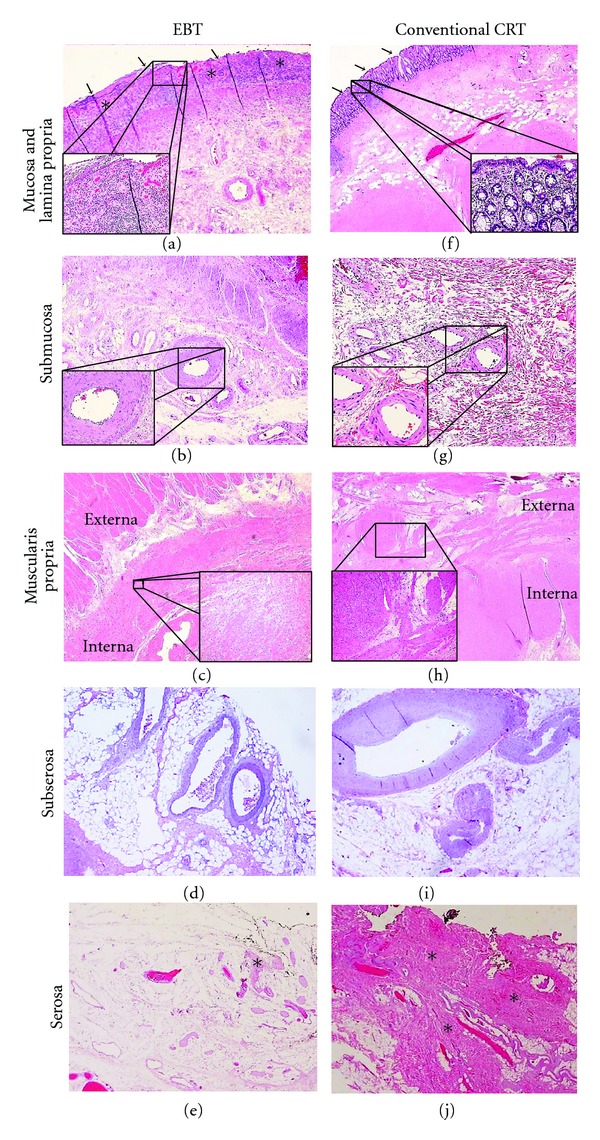
Representative H&E stained histopathologic sections at 4x magnification from patients who exhibited a complete pathologic response to EBT (a)–(e) and conventional external beam radiation (f)–(j). All images are taken from the region of the rectal wall where the tumor was located prior to neoadjuvant therapy. All insets are shown at 20x magnification. First row: at the mucosa, extensive ulceration (solid arrows) is apparent after EBT (a), while the mucosa remains intact (solid arrows) after conventional CRT (f). Hyalinization of the lamina propria (asterisks) is also evident after EBT (a). Second row: in the submucosa, marked hypertrophy and sclerosis of vessel walls can be seen following EBT (b), while only slight hypertrophy of vessel walls is seen after conventional CRT (g). Third row: within the muscularis propria, the more superficial interna layer can be seen to exhibit degeneration and atrophy after EBT while the externa layer remains largely intact (c); in a contrary fashion, following conventional CRT, it is the externa layer that exhibits more prominent degeneration compared to the interna (h). fourth row: at the level of the subserosa, vessel walls appear normal in patients treated with EBT (d), but distinctly hypertrophied in patients treated with conventional CRT (i). Fifth row: the serosa demonstrates few adhesions (asterisk) after treatment with EBT (e), in contrast to the extensive adhesions (asterisks) present after treatment with conventional CRT (j).

Surgical pathology slides from 5 cases in each of the 3 treatment groups (15 cases total) were randomly selected for rereview by a blinded pathologist to assess for qualitative differences in microscopic treatment effects. The surgical resection specimens showed disruption of tumor architecture (regions of necrosis and the presence of mucinous pools with sparse floating tumor cells) and radiation treatment effects for all 3 radiotherapy techniques. However, the distribution and degree of radiation-induced changes throughout the layers of the rectal wall were different for EBT versus conventional 3DRT/IMRT (since radiation treatment effects were found to be virtually identical between specimens from the 3DRT and IMRT groups, these groups will be collectively referred to as the conventional external beam group from this point forward). Compared to the conventional external beam group, specimens from EBT patients demonstrated more pronounced radiation-induced changes to the superficial layers of the rectal wall (mucosa, lamina propria, submucosa, and muscularis interna) in the region where the tumor was located (Figure 3). The mucosa overlying the tumor was observed to be ulcerated and the underlying lamina propria manifested extensive fibrosis and hyalinization (Figure 3(a)). The vessels in the submucosa were seen to have a thickened and sclerosed vascular smooth muscle layer (Figure 3(b)), but deeper vessels located in the subserosa were largely spared (Figure 3(d)). Atrophy, disorganization, and degeneration were evident in the muscularis propria interna, but to a lesser degree in the deeper muscularis propria externa (Figure 3(c)). Few serosal adhesions were observed.

In specimens from patients treated with conventional external beam radiation, pathologic findings were similar in nature, but opposite in distribution with deeper layers of the rectal wall being more prominently affected than more superficial layers (Figure 3). Ulceration of the mucosal surface overlying the tumor was milder (Figure 3(f)); the muscularis propria externa rather than interna exhibited more extensive degeneration (Figure 3(h)); deeper vessels near the serosa (Figure 3(d)) were more affected than submucosal vessels (Figure 3(g)); more numerous serosal adhesions were present (Figure 3(j)). These contrasting distributions of treatment effect suggest that EBT imparts a more intense ablative effect to the tumor and rectal tissue immediately surrounding it, while conventional external beam treatment generates more diffuse ablative effects throughout all layers of the rectal wall. These patterns are consistent with the different modes of radiation delivery represented by EBT and conventional external beam radiotherapy.

## 4. Discussion

 The current standard of care for locally advanced resectable adenocarcinoma of the rectum (AJCC stage II-III) is neoadjuvant chemoradiation with a 5-FU-based regimen, followed by total mesorectal excision and adjuvant FOLFOX chemotherapy [9]. Conventional neoadjuvant chemoradiation has shown improved local control but not survival compared to surgery alone [3]. Neoadjuvant chemoradiation results in downstaging of tumors with 8–16% of patients achieving a pathologic complete response (pCR) [9, 10, 14–16]. Patients that achieve pCR due to neoadjuvant chemoradiation have improved disease-free and overall survival [10, 17–22]. However, acute grade 3 and 4 toxicities associated with this treatment are seen in up to 30% of patients [9, 10, 15, 16]. To further improve pathological response rates and systemic disease control, additional chemotherapy agents, including oxaliplatin or irinotecan, were given with 5-FU based chemotherapy and concurrent radiation. This resulted in minimal improvement in pCR (15–20%) and often increased grade 3 and 4 acute toxicity [9, 10, 15, 16]. With the advent of total mesorectal excision (TME), local recurrence rates have decreased from 25–30% to 6–12% [23, 24]. As a result, some have questioned whether it is still necessary to treat all locally advanced rectal cancer patients with pelvic radiation.

 One of the main goals of rectal cancer treatment is sphincter preservation to maintain normal bowel function. Sauer et al. found that neoadjuvant conformal chemoradiation results in increased rates of sphincter preservation; however, long-term studies have demonstrated an overall decline in anorectal function [9]. For this reason, efforts have been made to limit the radiation dose to normal rectum and surrounding organs at risk (OARs) including the bladder and reproductive organs. Three-dimensional conformal radiation therapy (3DRT) seeks to accomplish this through the use of multileaf collimators and 3 to 5 beams in order to shape the radiation delivered to fit the profile of the target tumor. Intensity modulated radiation therapy (IMRT) utilizes a greater number of radiation beams (typically 5 to 9) to spare organs through a wider distribution of dose and more precise targeting of the rectal tumor plus a margin. While both 3DRT and IMRT attempt to decrease radiation dose to normal structures, they require an additional 2-3 cm margin in order to cover microscopic extension (clinical target volume; CTV) and account for set up error as well as rectal motion (planning target volume; PTV) [4, 6, 8]. Similar to 3DRT, IMRT requires 5-6 weeks of radiation with concurrent chemotherapy and is substantially more expensive than conformal radiation. It still remains to be determined whether IMRT confers a significant improvement in toxicity rates and quality of life relative to 3DRT.

High-dose-rate (HDR) endorectal brachytherapy (EBT) is a possible alternative to conventional external beam radiation. It has been used in various malignancies (prostate, head and neck, uterine, cervical, vaginal) to deliver high doses of radiation to the tumor over a short period of time. HDR EBT delivers endoluminal radiation to the mucosal surface overlying the rectal tumor in four fractions of 6.5 Gy (26 Gy total) over one week. Its rapid dose fall off limits the exposure of the normal surrounding tissues to radiation (Figure 1), thereby reducing treatment-related toxicities [4, 6, 8]. The advantages of HDR brachytherapy relative to low-dose-rate permanent implants include decreased geometric uncertainties arising from edema resolution and seed migration as well as the ability to tailor dose delivery by use of specific dwell times [4, 6, 8]. Compared to 3DRT and IMRT, HDR brachytherapy requires smaller margins (CTV/PTV expansion = approximately 1 cm for EBT) around the tumor, which allows greater sparing of organs at risk [8, 25]. The benefits of EBT include high tumor response rates and reduced cost relative to 3DRT and IMRT without the need for concurrent systemic chemotherapy and its associated toxicities [4, 6, 8, 25]. Other benefits of EBT are the short duration of treatment and decreased time to surgery. EBT planning takes less than a day while 3DRT and IMRT typically require 1-2 weeks for treatment planning. On average, patients receiving EBT will undergo surgical resection and receive adjuvant chemotherapy 5 weeks earlier than with conventional treatment [4]. Our study showed an even greater decrease in time to surgery, with patients who underwent EBT undergoing surgery approximately 7 weeks sooner than their 3DRT and IMRT counterparts.

There is limited data on the clinical outcomes and therapeutic benefits of EBT. Data on high-dose-rate brachytherapy for rectal cancer has only been published from one institution (McGill University in Montreal) by Vuong et al., who has treated over 300 patients with 29% achieving a pCR and 37% with only microscopic disease at the time of resection, while less than 1% experience acute grade 3 to 4 toxicities [4]. Estimated local recurrence rate is 5%, which is comparable to the standard of care [9]. Importantly, nodal recurrence was observed to be low with EBT and disease-free survival and overall survival were similar to historical controls. While encouraging, these results have not been externally validated. Preliminary results of the first 7 patients enrolled on a prospective EBT pilot study at Johns Hopkins Hospital documented here show similar results to the Montreal study. All patients had tumors less than 12 cm from the anal verge, no clinical/radiographic suspicious lymphadenopathy outside the mesorectum, and T2-T3/N0-N1 stage tumors. There was a trend towards a higher pCR rate in EBT patients with 43% found to have no residual tumor at time of surgery, compared to 14% of patients treated with IMRT and 7% of patients treated with 3DRT. The pCR rate for EBT observed in our study (43%) was similar to the 29% observed by Vuong et al. [4]. All patients treated with EBT in our study had negative margins at resection, and 86% were able to undergo a sphincter preserving surgery (lower anterior resection). Toxicity was less for EBT compared to conventional methods and was rare at a grade 3 or 4 level, as seen in the McGill data. Overall survival and progression-free survival for EBT were noninferior to conventional chemoradiation; however, with the small sample size and short median followup, definitive conclusions regarding survival outcomes cannot be drawn.

Radiologic analysis according to RECIST showed similar tumor response rates in EBT, 3DRT, and IMRT patients. Radiologic complete response (rCR) was observed in 1 patient in the EBT and IMRT groups, while no patient in the 3DRT group had an rCR. Interestingly, pathologic complete responses (pCR), defined as absence of any residual tumor cells, occurred at higher frequency than rCR in all 3 groups, with 3 EBT patients, 2 IMRT, and 1 3DRT patient manifesting pCR at surgery. Of the 3 patients who demonstrated pCR in the EBT group, 1 had an rCR, 1 a radiologic partial response (rPR), and 1 had radiologically stable disease (rSD) (Figure 2). This suggests that lack of rCR following neoadjuvant EBT does not rule out pCR. These findings agree with a study performed by Branagan et al., which found that preoperative radiologic rectal tumor staging using MRI showed a poor correlation (Kappa statistic = 0.18) with pathologic tumor stage of the resected specimen [26]. As microscopic pathologic examination of the TME specimen is the gold standard for assessment of tumor response, our data indicate that the radiologic response on preoperative MRI cannot be reliably used to predict degree of tumor response to EBT because even rSD can correlate with a pCR at resection. Positron emission tomography (PET) imaging may represent a more effective way to radiologically evaluate tumor response prior to surgery. Although a study performed at Stanford University showed that changes seen on PET have limited value in predicting for pathologic response of rectal cancer after conventional neoadjuvant chemoradiation, the utility of PET has not yet been examined in assessing tumor response to EBT [27].

One concern in treating rectal tumors with EBT instead of conventional external beam radiation is lack of sterilization of pelvic lymph nodes as a result of the rapid dose falloff associated with EBT, which covers only the mesorectal lymph nodes with little to no coverage of pelvic nodes. Thus, it is conceivable that EBT may lead to higher lymph node metastasis rates and local recurrence. For that reason, patients are selected for EBT based on pathologic lymph node status by imaging. Patients are excluded if positive lymph nodes are identified in the pelvis outside the mesorectum prior to treatment. It is encouraging that in our study preoperative MRI showed no development of pelvic node involvement for any of the 7 patients who received EBT. Furthermore, the one patient in the EBT group with radiologic involvement of mesorectal lymph nodes on pretreatment MRI exhibited complete resolution of nodal involvement on post-EBT imaging. Although limited in their generalizability by the small sample size, these findings suggest that when patients are carefully selected for neoadjuvant EBT (i.e., N0-N1 patients only), there is a low likelihood that they will develop radiologic evidence of N2 disease prior to surgery. This evidence confirms data presented by Vuong et al. documenting a 5-year local recurrence rate of 5% in N0-N1 patients treated with EBT, which likely indicates a 5% or lower rate of spread to pelvic lymph nodes prior to surgery [4]. However, longer followup and a greater number of patients are needed in our study before radiologic results regarding development of N2 disease prior to surgery can be correlated with local recurrence rates.

Radiation-induced injury to the rectum is well documented and characteristic histologic changes include architectural disruption and atrophy, goblet cell loss, shortened crypts, a thickened and distorted muscularis, intestinal wall fibrosis, serosal thickening, and vascular sclerosis [28, 29]. A study in mice that documented the histopathologic characteristics of radiation injury to intestinal tissue observed similar findings as those listed above [30]. Mice that received external beam radiotherapy showed mucosal ulcerations, fibrotic changes, serosal thickening, and marked vascular sclerosis. Effects on rectal tissue due to high-dose-rate (HDR) brachytherapy have not yet been published. However, an autopsy study evaluating the histological findings in prostate tissue treated with low-dose-rate brachytherapy showed similar results to our study [31]. The prostate specimens showed distorted glandular architecture, extensive fibrosis, and hyalinization of the blood vessels.

 Our study is the first to describe the histologic differences in treatment effect of EBT compared to conventional external beam radiation seen on pathologic examination of rectal adenocarcinoma resection specimens. In general, the types of histologic changes induced by EBT and conventional external beam radiation were similar, consisting of mucosal ulceration, fibrosis and hyalinization of the lamina propria, degeneration of the muscularis propria, and vessel wall hypertrophy and sclerosis as well as formation of serosal adhesions. Notably, however, the distribution and degree of these changes throughout the layers of the rectal wall were distinct for EBT. TME specimens from patients who received conventional external beam radiation demonstrated moderate radiation-induced changes diffusely throughout the rectal wall. Specimens from patients treated with EBT, on the other hand, displayed these changes along a gradient, with intense treatment effect apparent in the superficial layers of the rectal wall (mucosa, lamina propria, submucosa, and muscularis propria interna), but progressively reduced treatment effect in each of the deeper layers (muscularis propria externa, subserosa, and serosa). These contrasting distributions of treatment effect suggest that EBT may achieve a more potent localized ablative effect on the tumor and immediately surrounding rectal tissue than does conventional external beam radiation, but may not be as effective in sterilizing the serosa.

It follows that careful patient selection is critical for successful implementation of EBT. Patients with T1–T3 lesions may derive considerable benefit from the high ablative potential of EBT and would be considered viable candidates because their tumors can be adequately covered with EBT without extreme doses to the rectal wall. If tumors are more than 3-4 cm from the rectal wall, EBT may cause increased proctitis. Our results, as well as the data reported by Vuong et al. [4], indicate that EBT likely achieves higher pCR rates than conventional external beam radiation. A growing body of evidence supports the notion that patients with pCR after neoadjuvant therapy have more favorable long-term outcomes compared to patients with lesser or no pathologic response [10, 17–22]. Thus, it may be possible to improve outcomes in patients with T1–T3 rectal tumors by treatment with EBT rather than conventional external beam radiation. Patients with T4 lesions, however, may have portions of tumor that extend beyond the effective range of the radioisotope used in EBT and are likely better suited to conventional external beam radiation, which we have observed in this study to affect all layers of the rectal wall, including the outermost serosa.

Modern staging performed with endorectal ultrasound (EUS) and pelvic MRI has been shown to attain a high degree of accuracy in determining the T stage of rectal tumors. EUS has demonstrated a sensitivity of 90% and specificity of 75% for identifying T3 tumors, while MRI has a sensitivity of 80–86% and specificity of 71–76% [32–35]. In predicting adjacent organ invasion (T4 tumor stage), EUS and MRI have demonstrated sensitivities of 70% and 74%, respectively, and high specificity at 97% and 96% [33]. The use of these staging modalities has become routine in recent years as part of the workup for rectal cancer and can be utilized to discern patients well suited to EBT versus conventional external beam radiation in the clinical setting.

Finally, the limited range at which radiation treatment effects were observed for EBT on histopathologic examination in our study provides a rationale for the lesser degree of toxicity experienced by patients in the EBT group. Sauer et al. reported a 27% incidence of grade 3 to 4 acute toxicity as a result of neoadjuvant chemoradiation for rectal cancer [9]. More recent studies involving the addition of agents such as oxaliplatin to neoadjuvant chemoradiation regimens have been associated with rates of grade 3 to 4 acute toxicity as high as 36% [10–13]. The rate of acute toxicity in our study was well below this, with only one patient in the EBT group (14%) experiencing grade 3 proctitis. Thus, in addition to increasing the likelihood of achieving a pathologic complete response, EBT may also provide a less toxic mode of neoadjuvant therapy that appears at least as effective as long-course conventional chemoradiation as measured by short-term outcomes.

**Study Limitations**

Our study was primarily limited by a small number of patients and a short period of followup. These limitations precluded definitive survival analysis, but did not hinder evaluation of several clinical, radiologic, and pathologic outcomes of interest. The fact that only data on EBT patients was collected prospectively, while data on 3DRT and IMRT patients was collected retrospectively, introduces the biases inherent in retrospective studies to our analysis. It is also possible, though unlikely, that the difference in gender distribution between the EBT, 3DRT, and IMRT groups could confound our analyses, especially in regard to toxicity considering the different organs at risk in the pelvic region between males and females. Further followup of the EBT patients included in this study, as well as future EBT patients (trial enrollment goal is 30 patients), will be needed to determine median overall survival and thus estimate the impact of EBT on this primary oncologic outcome.

## 5. Conclusions

Comparison of preliminary EBT trial data to historical controls treated with conventional external beam radiation reveals that patients treated with EBT experience less toxicity and shorter time to surgery without compromising margin or lymph node status at resection. Followup was not sufficient for survival analysis, but EBT appears noninferior to 3DRT and IMRT at 6 months. EBT alone administered over 4 days achieves similar radiologic and favorable pathologic tumor response rates when compared to 5-6 weeks of conventional chemoradiation. EBT showed a more intense local ablative effect on histopathologic examination, suggesting a greater likelihood of achieving pathologic complete response and, consequently, improved long-term outcomes. Furthermore, radiation-induced changes due to EBT were tightly localized to the area of the tumor with greater sparing of normal tissues including small bowel, likely explaining the lower rate of toxicity observed in comparison to 3DRT and IMRT. Careful patient selection using EUS and MRI is necessary to ensure that patients with T4 tumors that extend beyond the range of the radioisotope used for EBT are not offered this therapy. In summary, EBT appears to be a promising mode of neoadjuvant treatment for low lying rectal adenocarcinoma. Longer followup and a larger multicenter study are needed to conclusively evaluate the potential of EBT to produce a survival benefit in this patient population.

## Figures and Tables

**Table 1:** Demographic and baseline disease characteristics for patients broken down by EBT, 3DRT, and IMRT groups with statistical comparison. EBT: endorectal brachytherapy; 3DRT: 3D conformal radiotherapy; IMRT: intensity-modulated radiotherapy; ECOG: eastern cooperative oncology group performance status; RT: radiotherapy; CEA: carcinoembryonic antigen.

Characteristic	EBT (*n* = 7)	3DRT (*n* = 14)	IMRT (*n* = 11)	*P*
Age (mean ± SD)	60.4 ± 17.4	58.2 ± 12.0	52.3 ± 7.6	0.32
Sex (female%)	100	29	64	0.007
Race (Caucasian%)	85.7	64.2	63.6	0.55
ECOG (mean ± SD)	0.21 ± 0.41	0.17 ± 0.39	0.20 ± 0.42	0.84
Pre-RT CEA (median (range) ng/mL)	4.5 (1.5–15.5)	7.4 (1.5–168.1)	3.7 (1.5–11.9)	0.35^∗^
Pre-RT tumor volume (median (range) cm^3^)	13.1 (0.9–26.4)	25.2 (6.3–119.0)	6.1 (1.9–76.6)	0.11^∗^
Time between pre-RT MRI and RT start (mean ± SD days)	20 ± 11	22 ± 10	34 ± 31	0.30
Number. T3 (%)	5 (71)	12 (86)	8 (73)	0.66
Number T4 (%)	0 (0)	2 (14)	0 (0)	0.25
Number N0 (%)	4 (57)	4 (29)	3 (27)	0.36
Number N1 (%)	3 (43)	8 (57)	6 (55)	0.82
Number N2 (%)	0 (0)	2 (14)	0 (0)	0.25
Distance of tumor from anal verge (mean ± SD cm)	6.2 ± 1.9	8.4 ± 5.0	5.4 ± 2.5	0.80

^∗^Medians and ranges are given to better represent the data, but statistical comparison was performed among means.

**Table 2:** Clinical outcomes broken down by EBT, 3DRT, and IMRT groups with statistical comparison. EBT: endorectal brachytherapy; 3DRT: 3D conformal radiotherapy; IMRT: intensity-modulated radiotherapy; RT: radiotherapy; CEA: carcinoembryonic antigen.

Clinical outcome	EBT (*n* = 7)	3DRT (*n* = 14)	IMRT (*n* = 14)	*P*
Time to surgery from RT start (mean ± SD days)	53 ± 8	104 ± 21	119 ± 51	<0.001
Time to surgery from RT end (mean ± SD days)	50 ± 8	65 ± 20	79 ± 51	0.038
Post-RT CEA (median (range) ng/mL)	3.2 (1.1–18.3)	3.9 (1.4–67.8)	1.9 (0.5–12.3)	0.41^∗^
Change in CEA pre-RT → post-RT (median (range) %)	−20 (−45 to +18)	−40 (−83 to +300)	−12 (−91 to +20)	0.36^∗^
No. with grade 1 toxicity (%)	4 (57)	14 (100)	9 (82)	0.025
No. with grade 2 toxicity (%)	1 (14)	8 (57)	2 (18)	0.056
No. with grade 3 toxicity (%)	1 (14)	1 (7)	1 (9)	0.87
No. who underwent sphincter-preserving surgery (%)	6 (86)	13 (93)	10 (91)	0.87
No. with postoperative complications (%)	4 (29)	4 (36)	2/7 (29)	0.90
No. alive at 6 months post-RT/total (%)	7 (100)	14 (100)	11 (100)	1.0
No. with local recurrence at 6 months post-RT (%)	0 (0)	0 (0)	0 (0)	1.0
No. with distant metastasis at 6 months post-RT (%)	0 (0/7)	1 (7)	1 (9)	0.73

^∗^Medians and ranges are given to better represent the data, but statistical comparison was performed among means.

**Table 3:** Radiologic outcomes broken down by EBT, 3DRT, and IMRT groups with statistical comparison. EBT: endorectal brachytherapy; 3DRT: 3D conformal radiotherapy; IMRT: intensity-modulated radiotherapy; RT: radiotherapy; CR: complete response; PR: partial response; SD: stable disease; PD: progressive disease; LN: lymph nodes.

Radiologic outcome	EBT (*n* = 7)	3DRT (*n* = 10)	IMRT (*n* = 7)	*P*
Post-RT tumor volume (median (range) cm^3^)	1.0 (0.0–3.6)	3.8 (0.7–26.3)	0.4 (0.0–5.5)	0.16^∗^
Time between RT end and post-RT MRI (mean ± SD days)	35 ± 3	35 ± 7	36 ± 9	0.98
% decrease in tumor volume pre-RT → post-RT (median (range))	89 (38–100)	87 (16–96)	93 (66–100)	0.78
No. CR (%)	1 (14)	0 (0)	1 (14)	0.46
No. PR (%)	4 (57)	9 (90)	6 (86)	0.23
No. SD (%)	2 (29)	1 (10)	0 (0)	0.26
No. PD (%)	0 (0)	0 (0)	0 (0)	1.0
No. with clinically significant mesorectal LN before treatment (%) → no. after treatment (%)	1 (14) → 0 (0)	6 (43) → 3 (21)	1 (14) → 1 (14)	—
No. with clinically significant pelvic LN before treatment (%) → no. after treatment (%)	0 (0) → 0 (0)	4 (29) → 4 (29)	1 (14) → 0 (0)	—

^∗^Medians and ranges are given to better represent the data, but statistical comparison was performed among means.

**Table 4:** Pathologic outcomes broken down by EBT, 3DRT, and IMRT groups with statistical comparison. EBT: endorectal brachytherapy; 3DRT: 3D conformal radiotherapy; IMRT: intensity-modulated radiotherapy; RT: radiotherapy; CR: complete response; LN: lymph nodes.

Pathologic outcome	ERBT (*n* = 7)	3DRT (*n* = 14)	IMRT (*n* = 11)	*P*
No. with pathologic CR of primary tumor at surgery (%)	3 (43)	1 (7)	2 (18)	0.06
No. with positive margins at surgery (%)	0 (0)	1 (7)	0 (0)	0.47
No. with LN involvement at surgery (%)	3 (43)	8 (57)	4 (36)	0.57
No. with lymphovascular invasion (%)	1 (14)	1 (7)	2 (18)	0.60
